# A Way to Close the Loop: Physicochemical and Adsorbing Properties of Soybean Hulls Recovered After Soybean Peroxidase Extraction

**DOI:** 10.3389/fchem.2020.00763

**Published:** 2020-08-26

**Authors:** Maria Laura Tummino, Valentina Tolardo, Mery Malandrino, Razieh Sadraei, Giuliana Magnacca, Enzo Laurenti

**Affiliations:** ^1^Department of Chemistry, Università di Torino, Turin, Italy; ^2^Centre for Nanostructured Interfaces and Surfaces (NIS) and INSTM Reference Centre, Turin, Italy

**Keywords:** soybean hulls, adsorption, wastewater treatments, scrap reuse, metals

## Abstract

Soybean hulls are one of the by-products of soybean crushing and find application mainly in the animal feed sector. Nevertheless, soybean hulls have been already exploited as source of peroxidase (soybean peroxidase, SBP), an enzyme adopted in a wide range of applications such as bioremediation and wastewater treatment, biocatalysis, diagnostic tests, therapeutics and biosensors. In this work, the soybean hulls after the SBP extraction, destined to become a putrescible waste, were recovered and employed as adsorbents for water remediation due to their cellulose-based composition. They were studied from a physicochemical point of view using different characterization techniques and applied for the adsorption of five inorganic ions [Fe(III), Al(III), Cr(III), Ni(II), and Mn(II)] in different aqueous matrixes. The behavior of the exhausted soybean hulls was compared to pristine hulls, demonstrating better performances as pollutant adsorbents despite significant changes in their features, especially in terms of surface morphology, charge and composition. Overall, this work evidences that these kinds of double-recovered scraps are an effective and sustainable alternative for metal contaminants removal from water.

## Introduction

The origin and early history of soybeans are unknown, but some agronomic publications recorded origins of soybeans back to 2800 B.C. in China. Soybean (*Glycine max*) is an annual crop and today represents one of the major industrial and food crops grown in every continent (Bekabil, 2015), reaching a global production of over 360 million metric tons in 2018–2019 (USDA, 2019). Soybean hulls are one of the by-products of soybean crushing, a necessary step to produce soybean oil and meal (Poore et al., 2002; Scapini et al., 2018). Hulls represent around 8% (w/w) of the seed (Middelbos and Fahey, 2008; Robles Barros et al., 2020) and find application mainly in the animal feedstuff sector, due to their low nutrient value (Li et al., 2011; Balint et al., 2020; Robles Barros et al., 2020). When such use is not possible, hulls are burnt to recover heat or disposed in landfill as putrescible waste (Robles Barros et al., 2020). According to previous works, they are constituted by variable amounts of cellulose (38–51%), hemicellulose (20–25%), lignin (4–8%), pectin (4–8%), proteins (11–15%), minor components (fatty acids, waxes, terpenes, essential oils, aromatic compounds, residual sucrose), and a little fraction of ashes (Wartelle and Marshall, 2000; Rojas et al., 2014).

One of the potential uses of soybean hulls in bio-chemistry field is their treatment to extract the soybean peroxidase (SBP). This enzyme is quite similar in structure and properties to the well-known horseradish peroxidase (HRP), adopted in a wide range of applications such as bioremediation and wastewater treatment, biocatalysis, diagnostic tests, therapeutics, and biosensors (Lopes et al., 2014; Krainer and Glieder, 2015). Respect to HRP, SBP shows a higher stability and a lower susceptibility both to thermal and chemical inactivation, making it suitable for biotechnological applications (Ryan et al., 2006; Steevensz et al., 2014; Al-Maqdi et al., 2018; Bilal et al., 2018; Donadelli et al., 2018; Sadraei et al., 2019; Yang et al., 2019). In a previous study regarding the prospects for a large-scale soybean peroxidase commercialization, Hailu et al. (2010) suggested that investments in an SBP extraction facility can be economically advantageous, estimating that, in a 0.5 ha plant, 6.2 metric tons of hulls can generate 0.56 billion units of crude SBP with a total annual revenue of 5.1 millions of CAD$.

Another possible way to valorize the soybean hulls is their application as adsorbents of metal ions or organic molecules (among others, those indicated as Contaminants of Emerging Concerns) in polluted waters. The presence of these species in water bodies represents one of the most concerning environmental issues for their detrimental repercussions on aquatic organism, plants, human health, and climate changes (Inyinbor Adejumoke et al., 2018). Within the scientific community, many efforts have been devoted to the development of different methods to solve this problem. Adsorption on biomasses results one of the most convenient solutions for two aspects: the adsorption does not favor secondary pollution if used with organic contaminants (i.e., transformation of the toxic substances into other kinds of polluting products) and it is a suitable method for capturing metal ions. In addition, the employment of residual biomasses is a key factor in a perspective of recycle and reuse. Carbon-based compounds, such as activated carbons, graphene, or graphene-oxides (De Gisi et al., 2016; Wang et al., 2018; Ali et al., 2019), and (hydro)oxide-based materials like SiO_2_, Al_2_O_3_, zeolites, clays, etc. (Chen et al., 2017; Shi et al., 2020) have been widely described in the literature. More recently, supported humic-like substances, natural polysaccharides (as chitosan, alginate, starch, cellulose), but especially various types of agricultural/domestic scraps have been exploited for adsorption purposes (Dai et al., 2018; Singh et al., 2018; Tummino et al., 2019, 2020). In this context, soybean hulls, rich of hemicellulose and cellulose, containing oxygenated functional groups including carbonyl groups, hydroxyl groups, and ethers, can bind heavy metal ions and organic pollutants by different kinds of interaction (chelation, complexation, coordination, formation of hydrogen bonds). A short, but representative, list of substances removed by soybean hulls-based adsorbents, as reported in the literature, is shown in Table 1.

**Table 1:** Inorganic ions and organic substances removed by adsorption with soybean hulls.

**Substrates**	**References**	**Hulls pretreatments**
Zn(II), Cu(II), Ni(II)	(Marshall and Johns, 1996; Marshall et al., 1999)	Different washings and/or citric acid-modification
Pb(II)	(Li et al., 2011)	Citric acid-modification
Cr(VI)	(Sheng-quan et al., 2012)	No treatments
Hg(II)	(Rizzuti et al., 2015)	No treatments
Safranin T, Remazol brilliant blue R, direct violet 51	(Rizzuti and Lancaster, 2013)	No treatments
BF-4B reactive red dye	(Módenes et al., 2019)	No treatments
BF-5G reactive blue dye	(Honorio et al., 2016)	No treatments
Methylene blue	(Fieira et al., 2019)	No treatments
Hormones	(Honorio et al., 2019)	No treatments
Herbicides (Diuron and Hexazinone)	(Takeshita et al., 2020)	No treatments

Moreover, in a previous paper, Marshall and Wartelle (2006) carried out modifications to make hulls act as dual-functional ion exchange resins and enhance their adsorbing properties, imparting a specific surface charge by reaction with citric acid (negatively charged) or choline chloride (positively charged).

Soybean hulls have been also considered as source of carbon (obtained by either thermal or chemical transformations) for the production of micro-mesoporous adsorbents (Girgis et al., 2011), biofillers (Balint et al., 2020), and can potentially be employed in those fields where carbons are required as active substrates for electrochemistry, electronics and biomedicine (Thiha et al., 2019; Sun et al., 2020; Wang C. et al., 2020). Finally, also cellulose and other polysaccharides, constituting the lignocellulosic hull biomass and obtained after proper extraction processes (Camiscia et al., 2018; Wang S. et al., 2020), can find outlet in different branches of biotechnology (food, medicine, bioremediation, paper industry, etc.) and of biorefinery, since they can be converted to biopolymers, bioethanol or even to fuels with high commercial value (Cassales et al., 2011; Camiscia et al., 2018; Dall Cortivo et al., 2020; Wang S. et al., 2020).

Given the benefits of promoting soybean hulls revalorization, this study is framed in the context of circular economy, aiming to (i) recover and reuse soybean hulls at the very end of their lifecycle after being subjected to the treatments for SBP extraction; (ii) study the physicochemical properties and adsorbing features of the treated hulls, also in comparison with the untreated ones. For these purposes, soybean hulls were characterized and tested toward solutions of the following inorganic ions, Fe(III), Al(III), Cr(III), Ni(II) and Mn(II), in different aqueous matrixes, namely pure water, potable waters and landfill leachate.

## Materials and Methods

All the reagents were purchased from Merck Life Science S.r.l. (Italy) and used without further purification. All the experiments were performed in triplicate.

### Preparation

For the extraction of soybean peroxidase (SBP) (Tolardo et al., 2019), the seeds were peeled, and the obtained hulls were stored at −12°C until use. SBP was extracted and purified by a process based on a previously published method (Calza et al., 2016): 100 g of soybean hulls were ground in a mortar, added to 600 mL of phosphate buffer (0.025 M, pH 7) and left under stirring for 2 h at room temperature. Then, the hulls were separated from the solution by filtration with a cotton gauze and subjected to the same treatment until the filtrate gave a negative response to enzymatic activity test for SBP. The hulls were successively dried at room temperature, cooled by N_2_ at 77 K to favor their grinding and, then, homogenized in a mortar. Hulls not subjected to SBP extraction were homogenized in the same way and used as reference samples. In this paper, treated and untreated hulls were labeled SBH-A and SBH-B, respectively, where A and B stands for “After” and “Before” the extraction.

### Characterization

ζ-potential measurements were performed on a Zetasizer (Malvern Instrument, Malvern, UK). The ζ-potential values were measured using principles of laser Doppler velocimetry and phase analysis light scattering (M3-PALS technique). All the suspensions were prepared by dispersing 10 mg of powder in 20 mL of double distilled water. The pH values were adjusted in a range of 2–10 by addition of 0.1 M HCl or 0.1 M NaOH aqueous solutions.

Attenuated total reflectance Fourier transform infrared (ATR-FTIR) spectra (16 scans/spectrum, 4 cm^−1^ resolution) were collected using a Universal ATR Sampling Accessory assembled in a Perkin–Elmer Spectrum 100 Fourier transform infrared spectroscope.

Scanning Electron Microscopy (SEM) analysis was carried out using a ZEISS EVO 50 XVP with LaB_6_ source, equipped with detectors for secondary electrons collection and an Energy Dispersive X-ray Spectrometry (EDS) probe for elemental analyses. Samples were covered with a gold layer of ~15 nm of thickness before the analysis to prevent charging (Bal-tec SCD050 sputter coater).

Surface area and pore volumes were obtained by N_2_ adsorption at 77 K in an ASAP2020 gas-volumetric apparatus (Micromeritics, Norcross, GA, USA). The samples were previously outgassed overnight at 100°C until a standard residual pressure of 10^−2^ mbar was stably present in the outgassing system. The specific surface area of soybean hulls was calculated by the Brunauer–Emmett–Teller (BET) method (Brunauer et al., 1938).

The release of substances from SBH-A and SBH-B (1,600 mg L^−1^) in MilliQ® water at pH 5 and 7 was monitored. After stirring for 24 h, hulls were separated by filtration in a Büchner funnel and the amount of each metal ion in solution was determined by Inductively Coupled Plasma Optical Emission Spectrometry (ICP-OES), model Optima 7000 DV (Perkin Elmer, Waltham, MA, USA), equipped with a crossflow nebulizer, a Scott spray chamber and a double monochromator (prism and Echelle grating). The instrumental conditions were: plasma power 1.3 kW, sample aspiration rate 1.5 mL min^−1^, argon nebulizer flow 0.8 L min^−1^, argon auxiliary flow 0.2 L min^−1^ and argon plasma flow 15 L min^−1^. Moreover, in order to follow the simultaneous loss of organic substances, UV-visible spectra of the same solutions were recorded by an UV-visible spectrophotometer CARY 100 SCAN (Varian, Palo Alto, CA, USA) with a sample quartz cell of 1 cm path length.

### Adsorption/Desorption Experiments

Following a previously reported procedure for the adsorption of metallic ions (Tummino et al., 2019), aqueous solutions (75 mL) of Iron, Aluminum, Nickel, Manganese and Chromium ions, prepared by concentrated commercial standards Tritisol® in MilliQ® water [respectively, FeCl_3_, Al(NO_3_)_3_·9H_2_O, NiCl_2_, MnCl_2_, CrCl_3_], were put in contact at 25 ± 1°C with soybean hulls in a beaker and left under mechanical stirring throughout the measurement. During the experiments, pH and temperature were continuously monitored by means of a pH electrode and a thermometer introduced in the beaker. At different times, 10 mL of suspension were withdrawn and filtered with a cellulose filter (0.45 μm, Minisart, Sartorius, Göttingen, Germany) supported on syringes with plungers devoid of rubbery parts (BD DiscarditTM) to remove the adsorbent. After filtration, 10 μL of ultrapure HNO_3_ (65%, Suprapur®, Merck) were added to each sample and the solutions were stored at 4°C until further analysis. Inorganic ions concentration was determined by Inductively Coupled Plasma Optical Emission Spectrometry (ICP-OES), adopting the conditions previously described.

Initial tests were carried out by adding SBH-A or SBH-B at different concentrations (800 and 1,600 mg L^−1^) for 1 or 24 h to a solution containing all the metallic species: Fe(III), Al(III), Ni(II), Mn(II), and Cr(III) (1 × 10^−5^ M for each ion). The pH was modulated by adding NaOH or HNO_3_ solutions (0.2 M) in order to reach the stable pH value of 5, chosen after preliminary tests (not shown) to ensure the adsorption process without incurring precipitation problems. Then, for the most efficient system, namely SBH-A, the adsorption properties were studied more deeply in presence of: (i) solutions of a single inorganic ion (1 × 10^−4^ M) at pH 5; (ii) solutions of Cr(III) at different concentrations (from 1 × 10^−3^ to 2 × 10^−1^ mM) to construct the adsorption isotherm at pH 5 and 25°C; (iii) potable waters tested without any modification (pH 7.5); (iv) a landfill leachate tested without any modification (pH 5.6).

The experiments with potable waters and landfill leachate were performed for 6 h of contact time in order to optimize adsorption while maintaining the manipulation of the leachate within a typical working day (for safety reasons), in accordance with a previous procedure with similar samples (Tummino et al., 2019). The potable waters, obtained from different municipal wells, and the landfill leachate were supplied by Acea Pinerolese Industriale S.p.A., a waste treatment facility connected with a water depuration plant located in Pinerolo, Italy.

The desorption tests were performed on SBH-A, recovered by filtration in a Büchner funnel after a 24 h-adsorption experiment in presence of the mixed ions solution (ions concentration: 1 × 10^−5^ M for each ion and hulls concentration: 1,600 mg L^−1^). The desorption was conducted with two different solutions: (i) MilliQ® water at pH 5 and (ii) MilliQ® water at pH 5 containing NaCl salt in a 1:1 concentration ratio. The obtained suspensions were left under stirring for 24 h, then the hulls were separated from the solution by filtration. The ion concentrations in filtered solutions have been determined by ICP-OES.

## Results and Discussion

### Hulls Characterization

The treatment employed for SBP extraction clearly modified the macroscopic aspects of hulls. The most visible effect was the adhesion of the SBH-A hulls to each other, not observable in SBH-B. This characteristic was assessed by measuring the hulls thickness by a digital thickness gauge on a casual and representative set of samples before grinding: the thickness values were 100 ± 30 μm for SBH-B and 533 ± 160 μm for SBH-A.

In order to better define the surface and structural modification induced by the SBP extraction process, SBH-A samples were characterized by means of ζ-potential measurements, ATR-FTIR analysis and electron microscopy, whose relative results were compared with SBH-B properties.

#### ζ-Potential

**Figure 1:**
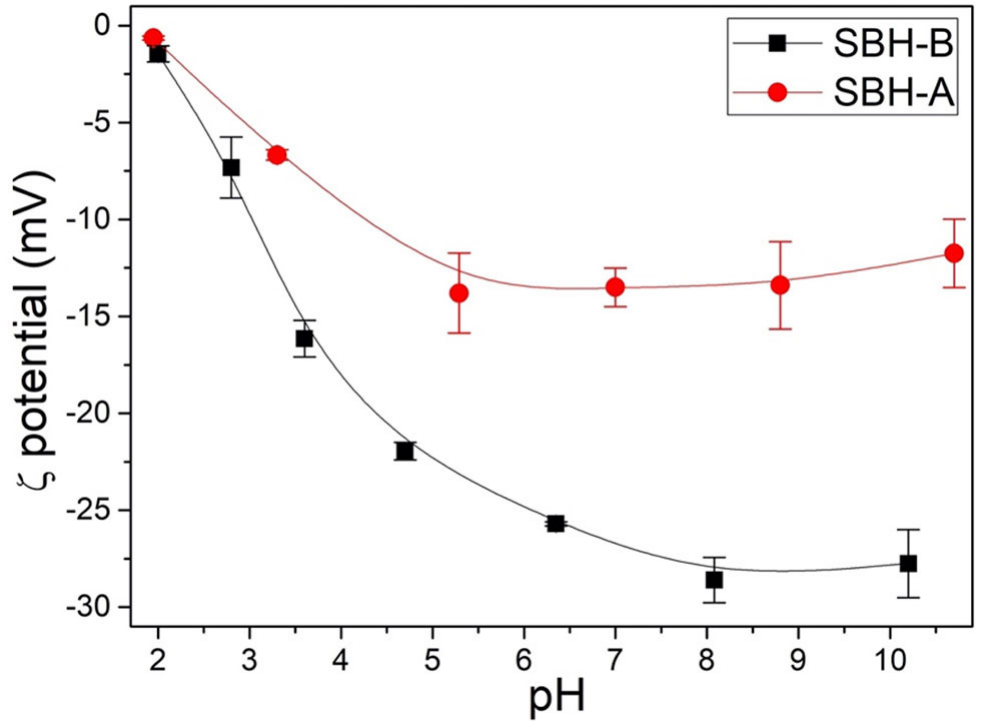
ζ-potentials of SBH-B and SBH-A at different pH values.

ζ-potential of SBH-A and SBH-B samples suspended in double distilled water were measured at different pH values. These measurements indicated that the surface of both the samples was always negatively charged also at acidic pH, approaching the point of zero charge at pH close to 2 (Figure 1). Moreover, the process for SBP extraction clearly influenced the surface of the hulls since the ζ-potential values of SBH-A are less negative than those recorded for SBH-B in the whole pH range, suggesting that the phosphate buffer is able in removing substances with a low pKa, which are negatively charged in a large range of pH values and therefore resulting more soluble than other not-charged substances (see release of organic matter in paragraph Release From SBH-A and SBH-B). The strong impact of this kind of pretreatment has been already ascertained by Giri et al. (2017) who observed the modification of physical structure and thermal stability of soybean hulls subjected to pyrolysis.

#### ATR-FTIR Measurements

**Figure 2:**
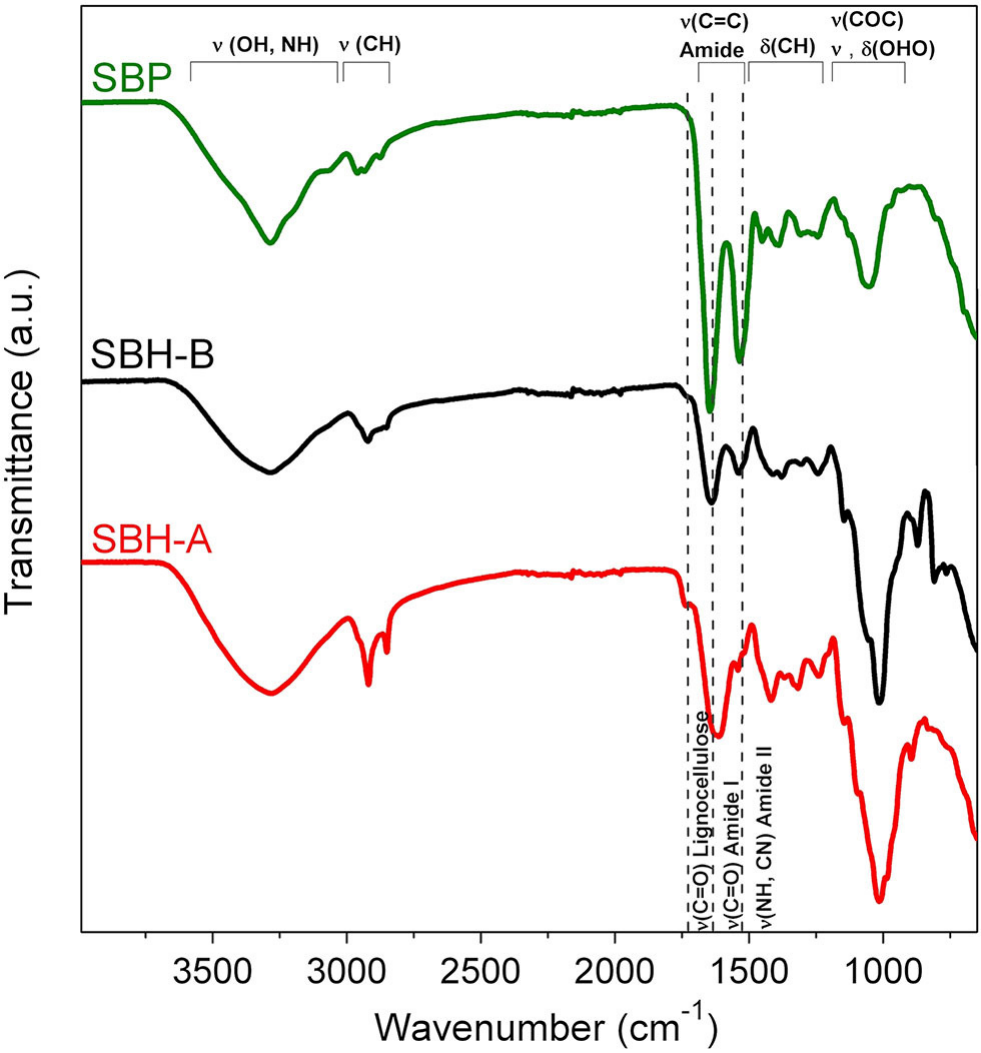
ATR-FTIR spectra of SBP commercial sample, SBH-B and SBH-A. Main vibrational modes observed in the spectra are indicated in the figure.

ATR-FTIR spectrum of pure SBP enzyme (Figure 2) displayed absorbance bands between 3,000 and 3,500 cm^−1^ attributed to the N–H and O–H stretching modes, respectively, whereas the C–H asymmetric stretching was observed around 2,930 cm^−1^. Bands at 1,645 and 1,530 cm^−1^ were ascribed to the amide I and amide II absorbance bands, respectively (Torres et al., 2017). The amide I band is mainly associated with the C=O stretching vibrations of the peptide bonds and it is closely correlated to the protein secondary structure, whereas amide II results from the N-H bending vibration and the C-N stretching vibration (Barth, 2007). The region between 1,200 and 1,400 cm^−1^ involved mainly C–H bending modes (Torres et al., 2017), whereas the signal at ca. 1,050 cm^−1^ was associated to O–H^+^–O stretching or bending of hydrated protons in proteins (Barth, 2007).

In general, ATR-FTIR spectra of hulls showed a broad band between 3,000 and 3,500 cm^−1^ of the N–H and O–H stretching modes, the peaks at 2,920 and 2,850 cm^−1^ were attributed to –CH_2_ asymmetric and symmetric stretching vibrations, respectively (Chandane and Singh, 2016). The peak at 1,740 cm^−1^ represents the carbonyl group (–C=O) stretching (Chandane and Singh, 2016), typical of ligno-cellulosic materials (Widiarto et al., 2019). The signal at 1,640 cm^−1^ (for SBH-B) and 1,620 cm^−1^ (for SBH-A) was associated to C=O stretching vibrations of the peptide bonds, if present, (Torres et al., 2017), to olefinic C=C stretching vibration (Qin et al., 2011) and to δ_HOH_ vibrations of molecularly adsorbed water (Widiarto et al., 2019). The peak at 1,540 cm^−1^ was due to N-H bending vibration and C-N stretching related to proteins (Torres et al., 2017) and/or to aromatic –C=C– stretching, which are vibrations mainly related to the ligno-cellulosic backbone (Chandane and Singh, 2016). At 1,420 and 1,370 cm^−1^ there are the regions of CH_2_ bending vibration and deformation of C-H in aromatic ring (Widiarto et al., 2019). The broad band centered at 1,010 cm^−1^ was assigned to ether (–C–O–C–) stretch (Chandane and Singh, 2016) and the peak at 870 cm^−1^ was attributed to glycoside bond of cellulose (Widiarto et al., 2019). The most remarkable differences between spectra of SBH-B and SBH-A concerned: (i) the peaks between 2,920 and 2,850 cm^−1^, sharper in the case of SBH-A; (ii) the intensity of the peak at 1,740 cm^−1^ of the carbonyl group (–C=O), stronger for SBH-A; (iii) the shift of the peak at 1,640 to 1,620 cm^−1^ and the decrease of the signal at 1,540 cm^−1^ in the case of SBH-A. These evidences suggested that the peptidic portion on the surface hulls was almost completely lost after the SBP extraction treatment, whereas the ligno-cellulosic backbone signals became prevailing.

#### Morphological Characterization

**Figure 3:**
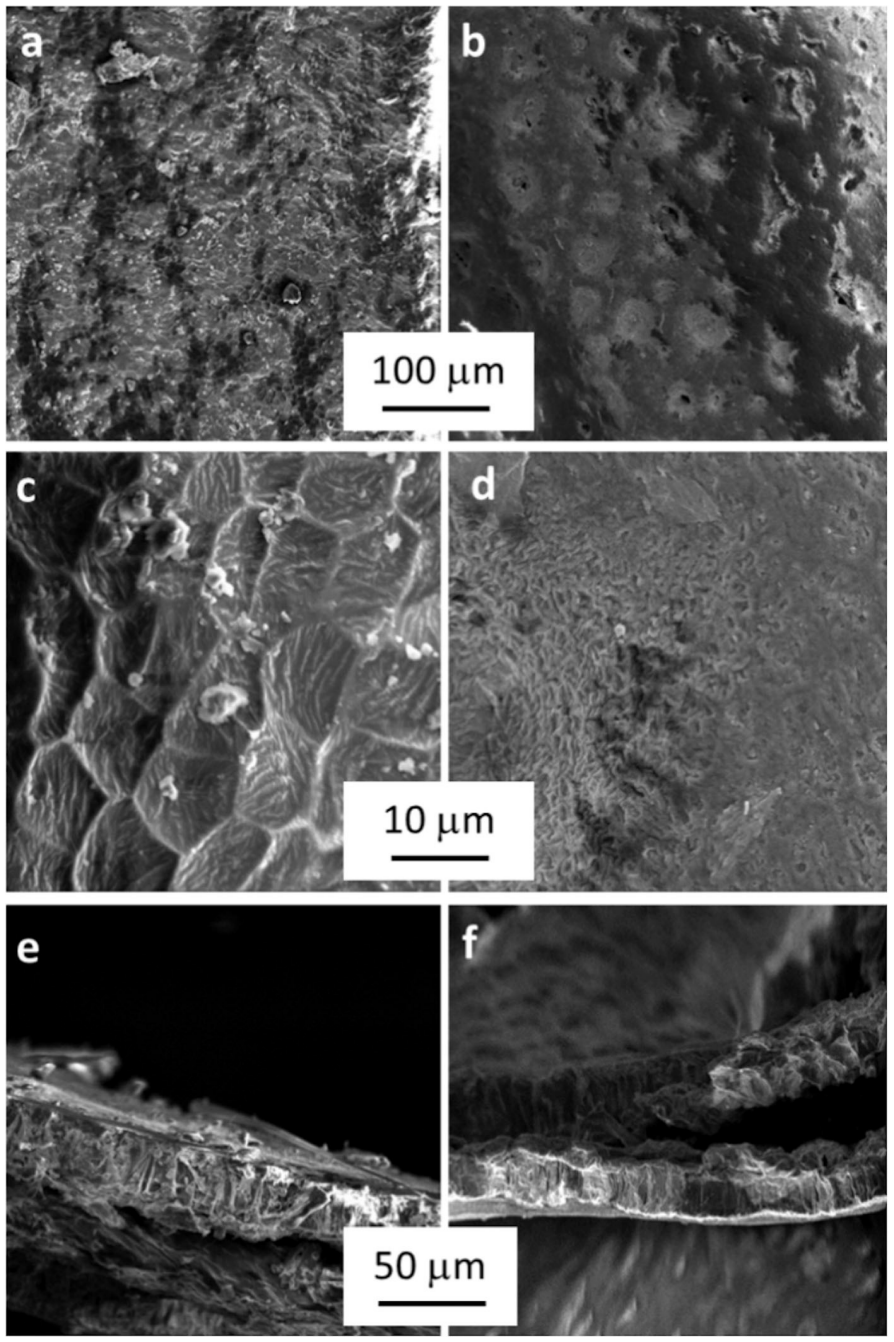
Micrographs of SBH-B **(a,c)** and SBH-A **(b,d)** surfaces at different magnifications and, at the bottom, pictures of SBH-B **(e)** and SBH-A **(f)** sections.

Nitrogen adsorption at 77 K revealed a very low surface area, <1 m^2^ g^−1^, for both treated and untreated samples. On the other hand, SEM micrographs (Figure 3) confirmed the surface modifications highlighted before showing evident morphological differences between SBH-A and SBH-B. Indeed, SBH-B had a rough surface with some cavities (Chandane and Singh, 2016) and scales with a diameter comprised between 5 and 10 μm. After the SBP extraction, the hull surface structure seemed to collapse, the section appeared more compact and the order constituted by the scales was lost.

#### Release From SBH-A and SBH-B

**Figure 4:**
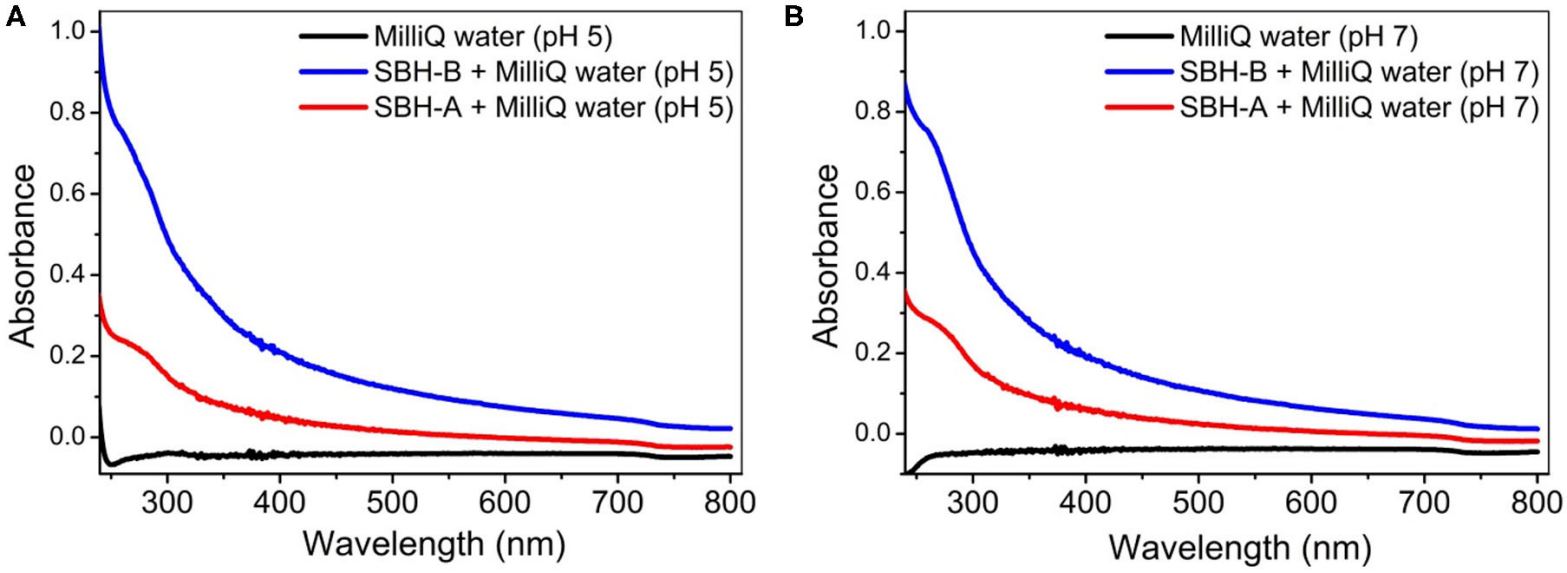
UV-visible spectra of the solutions obtained after the release tests of SBH-A and SBH-B (1,600 mg L^−1^) in MilliQ® water at pH 5 **(A)** and 7 **(B)**.

The release of the metal ions involved in this study was detected at pH 5 and 7 after the hulls were soaked in pure water for 24 h (Table 2), in order to probe the metal content which could interfere in the adsorption/desorption tests. The reproducibility of the results is confirmed by low percent relative standard deviation (RSD %) which was always <5%. SBH-B released a higher content of metals than SBH-A, confirming that SBP extraction procedure resulted in the removal of impurities present in the ligno-cellulosic structure of the hulls. Relevant amounts of iron, aluminum and, to a lesser extent, manganese were found in solution, in accordance to their ubiquitous presence as essential elements for living matter and their widespread diffusion in soils (Spehar, 1994; Noya et al., 2014). At increasing pH, the release process became less favorite. Nevertheless, in all cases, the amount of metals detected in the solution was lower than the concentration used in the adsorption tests to evaluate hulls' sequestrating capacity. The aqueous solutions were further analyzed by means of UV-vis technique, in order to follow the simultaneous loss of organic matter. The UV-visible spectra in Figure 4 show a non-negligible shoulder centered at 275 nm, indicating the release of water soluble organic substances (Khan et al., 2014), in particular in the case of SBH-B. A similar behavior have been already evidenced by Fieira et al. (2019), who evaluated such release in terms of Chemical Oxygen Demand (COD).

**Table 2:** Species released from SBH-A and SBH-B (1,600 mg L^−1^) in MilliQ® water at pH 5 and 7.

		**Species released (nM)**				
		**Fe**	**Al**	**Ni**	**Mn**	**Cr**
pH 5	SBH-B	994	320	8.0	30	2.9
	SBH-A	103	19	1.1	6.3	3.2
pH 7	SBH-B	572	188	13	19	1.8
	SBH-A	64	16	1.7	5.7	1.6

*Average errors (4–5%) are not shown in the table*.

An overall view of the characterization outcomes for SBH assesses a sort of cleaning effect of the protein extraction with phosphate buffer from inorganic and organic substances present in the main lignocellulosic structure. It is reasonable to image that, together with the loss of a part of hulls' mass, the interactions keeping together the lignocellulosic matter components were subjected to changes: in particular, several functional groups, initially interacting each other in the non-modified structure, remained isolated, and available to form other interactions with other substrates (in this case, metal ions). Simultaneously, the changes induced in the surface morphology can be ascribable to the swelling of the lignocellulosic matter and subsequent drying that, probably, caused a partial structure collapse (Fidale et al., 2008).

### Adsorption/Desorption Experiments

#### Adsorption of Metal Ions Mixture

**Figure 5:**
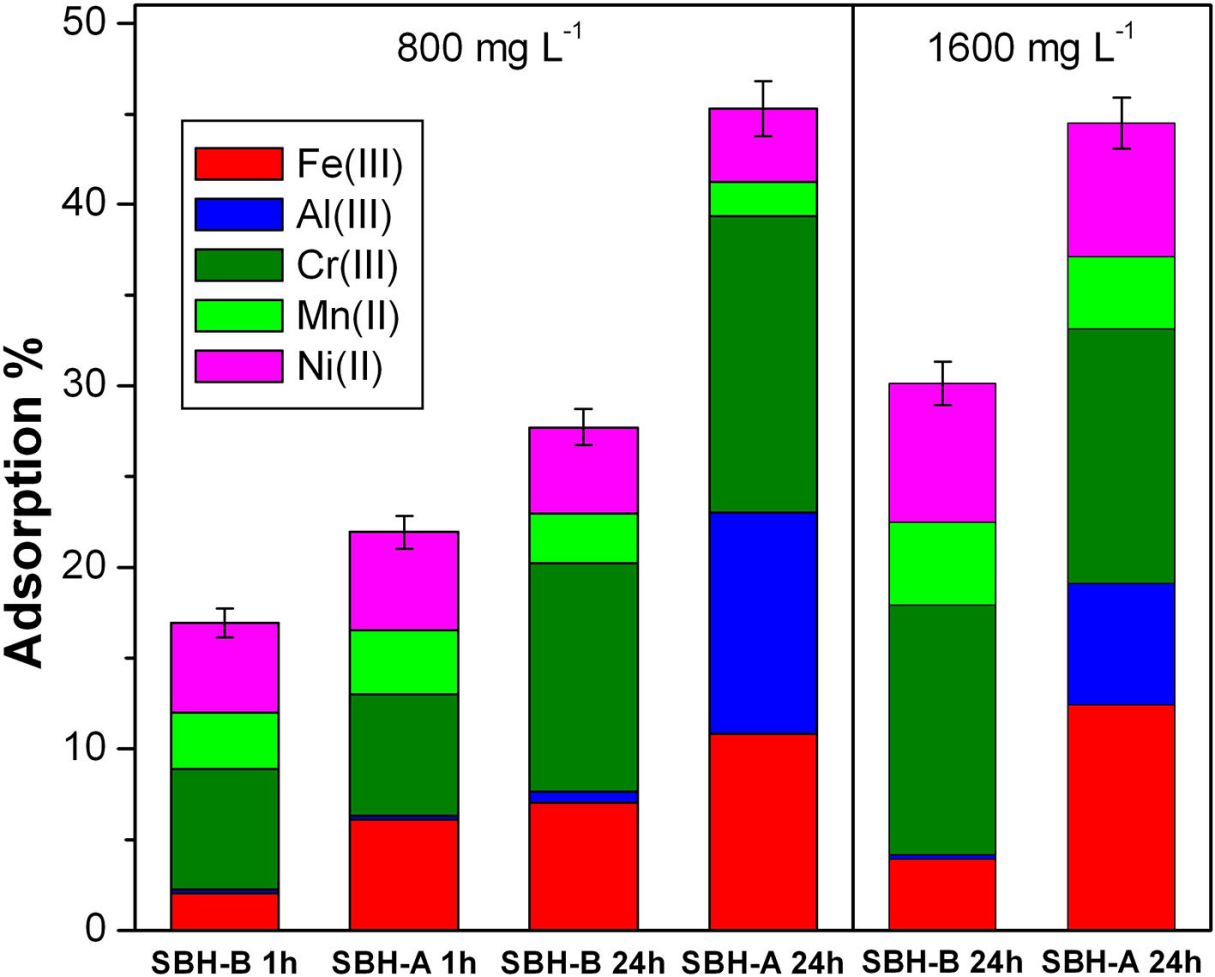
Adsorption % of Fe(III), Al(III), Cr(III), Mn(II), and Ni(II) mixed together (1 × 10^−5^ M for each ion) at pH 5 in presence of SBH-B and SBH-A; Left panel conditions: hulls concentration 800 mg L^−1^ and contact times 1 or 24 h; Right panel conditions: hulls concentrations 1,600 mg L^−1^, contact time 24 h. The adsorption % was calculated in relation to the total initial amount of metal ions in solution as described in the text.

SBH-A and SBH-B were tested for their adsorbing capability and the correspondent results are shown in Figure 5. Adsorption % was calculated by the following equation, where C_0_ is the starting concentration of each ion (1 × 10^−5^ M) and C_*ion*_ is the concentration of each ion left in the solution at the end of the experiment. The error was calculated with respect to the total amount of the adsorbed ions. To highlight the differences in the adsorption of different metal ions, the contribute of each ion was indicated in a proper color in Figure 5.

$$\begin{array}{l} Adsorption\;\%=\\ \frac{\left[C_{0}-C_{Fe\left(III\right)}\right]+\left[C_{0}-C_{Al\left(III\right)}\right]+\left[C_{0}-C_{Cr\left(III\right)}\right]+\left[C_{0}-C_{Ni\left(II\right)}\right]+\left[C_{0}-C_{Mn\left(III\right)}\right]}{5\times 10^{-5}}\times 100 \end{array}$$

Figure 5, left panel, displays the different performances of SBH-A and SBH-B (800 mg L^−1^) in contact with a solution containing the five metal ions (1 × 10^−5^ M for each ion) for two different contact times: 1 and 24 h. Many differences are highlighted from the experimental data, both concerning the adsorption properties of the sample before and after the SBP extraction, and the behavior toward the metal ions. Observing the trends related to iron adsorption, the use of SBH-A was evidently advantageous with respect to SBH-B sample, particularly after a longer contact time. Similarly, 1 day-contact favored the removal of Cr(III), mostly for SBH-A. In the cases of Ni(II) and Mn(II), the adsorption levels resulted comparable for SBH-A and SBH-B both after 1 and 24 h, whereas a peculiar behavior was demonstrated toward Al(III), which was captured significantly only by SBH-A after 24 h.

In the right panel of Figure 5, the degrees of adsorption reached after 24 h with 1,600 mg L^−1^ of hulls are represented. In general, the hull increment did not significantly affect the adsorption in the presence of the trivalent cations, whereas the adsorption observed for the two divalent cations, Mn(II) and Ni(II), almost doubled. A better adsorptive activity was once more achieved by SBH-A than SBH-B toward iron and aluminum ions.

From the interpretation of the two graphs of Figure 5, it can be noticed that, although the adsorption is generally a fast process, involving the adsorbent surface and multiple interactions, the simultaneous presence of different ions slowed down the process, establishing a certain selectivity, as well. The equilibria varied over time, favoring the species with a higher positive charge, Fe(III), Al(III) and Cr(III), possibly due to their electrostatic affinity with the negatively charged hulls surface. Nevertheless, the description of the adsorption phenomenon cannot be associated only to the electrostatic forces. Indeed, the hulls behavior toward aluminum resulted very peculiar: Al(III) has the lowest ionic radius (then, less steric hindrance) and a strong positive charge (3+), but it was not sequestrated by SBH-B and 1 h was not a sufficient contact time to allow an efficient adsorption. This occurrence can be justified by taking into account the solvation degree: smaller ions with high density charge are more solvated and less rapidly attracted by the adsorbent surface (Zhu et al., 2016). In general, a complex frame of factors connected to the adsorbate nature influences the adsorption effectiveness, as valence, electronegativity, hydration radii, hydration enthalpies, solubility of the cations (Zhu et al., 2016) and hard—soft, acid–bases affinity [according to Pearson's principle (Alfarra et al., 2004)]. Moreover, it is worth to underline that the modifications induced by SBP extraction, including a lowering of the surface's negative charge, did not compromise the adsorptive properties of hulls, but rather improved them in some cases. The most probable reasons are the lower competition with intrinsically present metal ions, which were mostly released during the SBP extraction treatment, and the exposure of a higher number of active sites on the surface of ligno-cellulosic matter after the same procedure. In particular, the SBH functionalities capable of positive ion attraction are mainly hydroxyl and carboxylic groups (Dai et al., 2018), which are present to a different extent in SBH-A and SBH-B, according to FTIR results.

#### Desorption Tests

Taking into account the adsorption capacity of SBH-A for most of the metals considered, the desorption tests were conducted on this material only. The hulls were recovered by filtration after 24-h adsorption experiment, the sample was divided in two aliquots and each successively added to a different solution, namely ultrapure water at pH 5 with or without NaCl. The intense interaction already found for hulls toward Al(III), Fe(III) and Cr(III) was responsible for the extremely reduced desorption observed for these ions, namely 1–3% maximum. Ni(II) and Mn(II) were slightly released, but always <20%. In general, the adsorption on SBH-A was not reversible in the adopted conditions, without any advantage provided by the presence of other metal ions (Na^+^) in the washing solution.

#### Adsorption of Metal Ions Separately

**Figure 6:**
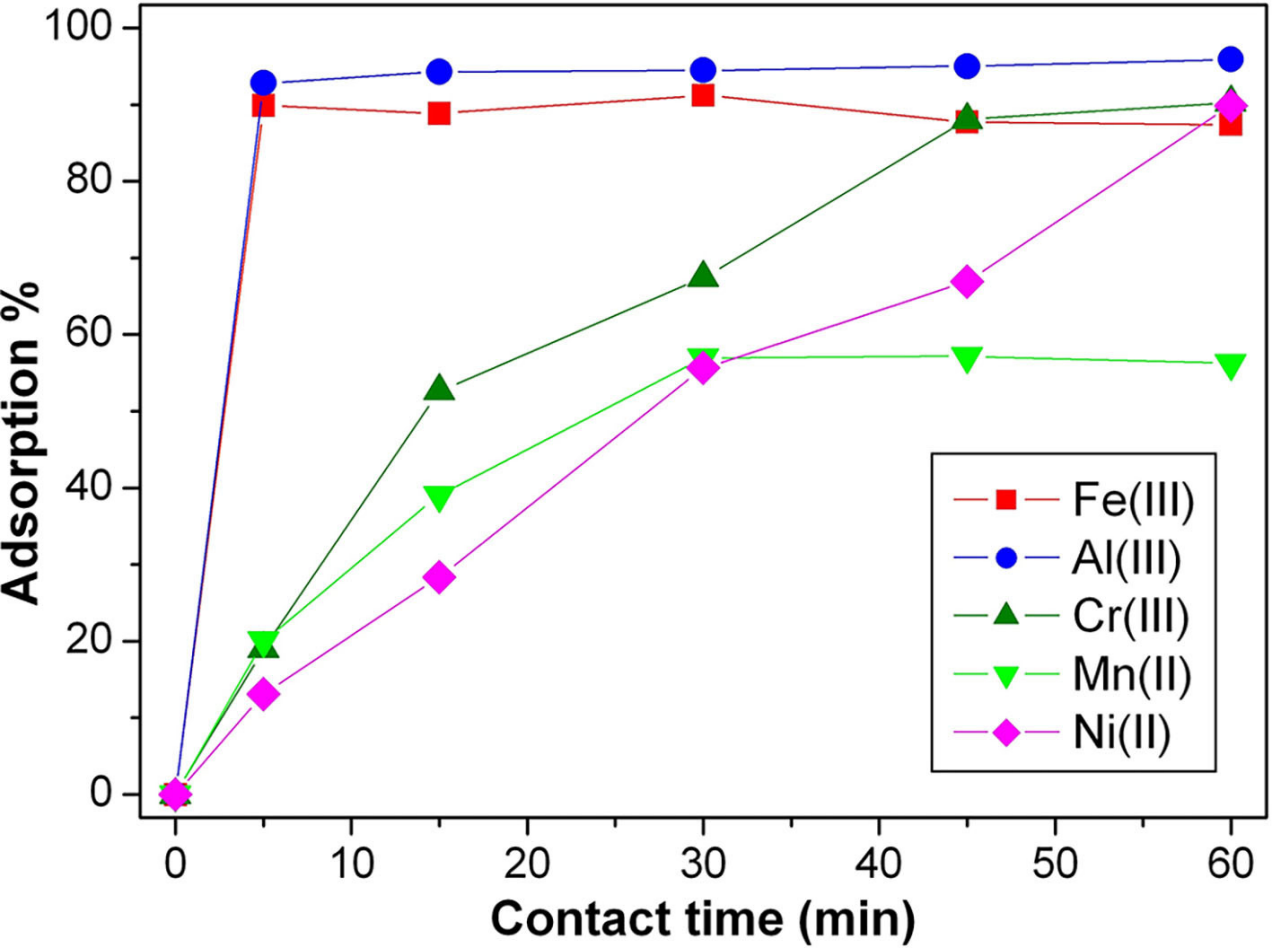
Adsorption kinetics of Fe(III), Al(III), Cr(III), Mn(II), and Ni(II), single ion solutions 1 × 10^−4^ M at pH 5, in presence of SBH-A (800 mg L^−1^).

Deepening the behavior of SBH-A, the hulls underwent adsorption of the same metal ions separately, using a concentration 10 times higher than the previous ones. As shown in Figure 6, high levels of metals removal were already reached in 1 h. Except for manganese, the entity of metals removal was ~90%, despite of different kinetic trends. In the absence of competition among different ions, the adsorption was favored for all the ions tested but the highly charged metal ions showed faster adsorption kinetic as confirmed by the first order *k*_*app*_, reported in Table 3. In particular, aluminum and iron ions show the highest *k*_*app*_ values and were almost totally adsorbed in few minutes, supporting the hypothesis that the present trials were mainly driven by charge interactions.

**Table 3:** First order kinetic constants calculated on the basis of single ion adsorption reported in Figure 6 (ion concentration 1 × 10^−4^ M at pH 5, in presence of SBH-A 800 mg L^−1^).

**Ion**	***k_***app***_* (min^**−1**^)**	***R*^**2**^**
Fe(III)	0.823 ± 0.281	0.998
Al(III)	0.765 ± 0.081	0.999
Cr(III)	0.047 ± 0.009	0.992
Mn(II)	0.082 ± 0.012	0.992
Ni(II)	0.012 ± 0.006	0.993

To get some preliminary information on the adsorption mechanism of SBH-A, Cr(III) was chosen as a representative ion to obtain an adsorption isotherm. In this case, the amount of adsorbed Cr(III) by SBH-A was measured after 24 h of contact between SBH-A and variable concentrations of Cr(III). Preliminary measurements confirmed that the equilibrium was reached at all the Cr(III) concentrations considered in the experiment.

The experimental data were fitted both with the Langmuir and Freundlich equations reported below:

$$\begin{array}{l} Langmuir:\frac{1}{q_{e}}=\frac{1}{q_{m\;}b{\;C}_{e}}+\frac{1}{q_{m}}\\ Freundlich:{Log\;q}_{e}={Log\;K}_{f}+\frac{1}{n}LogC_{e} \end{array}$$

In both the equations, *q*_*e*_ is the concentration of adsorbate on the solid and *C*_*e*_ is the concentration of Cr(III) at equilibrium. In the Langmuir equation, *q*_*m*_ is the sorption capacity (namely the amount of adsorbate at complete monolayer coverage) and *b* the Langmuir isotherm constant that relates to the energy of adsorption. In Freundlich equation, the value of *K*_*f*_ is indicative of the adsorption capacity and 1/*n* represents the adsorption intensity (Islam et al., 2013).

The parameters determined by fitting the experimental data with the two equations are reported in Table 4. The highest value of *R*^2^ obtained using the Langmuir model showed that it could be the most suitable model describing the system under study, indicating a homogenous distribution of adsorption sites and the presence of a single layer of Cr(III) ions on the surface of the hulls, rich of negatively charged functional groups (Saruchi and Kumar, 2019). On the other hand, from the Freundlich model, the value of 1/*n* value obtained in the range between 0 and 1, indicated that the interaction between Cr(III) and the hulls occurred easily (Saruchi and Kumar, 2019).

**Table 4:** Values of Langmuir and Freundlich constants, related to adsorption of Cr(III) ions on SBH-A (800 mg L^−1^) at pH 5, time of contact 24 h.

**Langmuir**		**Freundlich**	
*q_*m*_* (mmol g^−1^)	0.27	*K_*f*_* (L g^−1^)	11.2
*b* (L mmol^−1^)	129	1/*n*	0.94
*R*^2^	0.98	*R*^2^	0.88

#### Tests on Potable Waters and Landfill Leachate

On the basis of previous results, final trials were carried out on real potable waters and a landfill leachate. Such experiments were performed as described above and in accordance to a previously reported procedure with similar samples (Tummino et al., 2019). Other metal ions (zinc and lead) were also followed, albeit not considered in the previous tests, but present in the real water samples.

Potable waters from two different urban well-sources were tested: in both cases the presence of iron, aluminum and chromium species were negligible, whereas low concentrations of nickel, zinc, and lead (respectively, 0.03, 0.61, and 0.006 μM) were observed. Adsorption test with SBH-A led to the removal of 63% of Ni, 85% of Zn and 41% of Pb.

**Figure 7:**
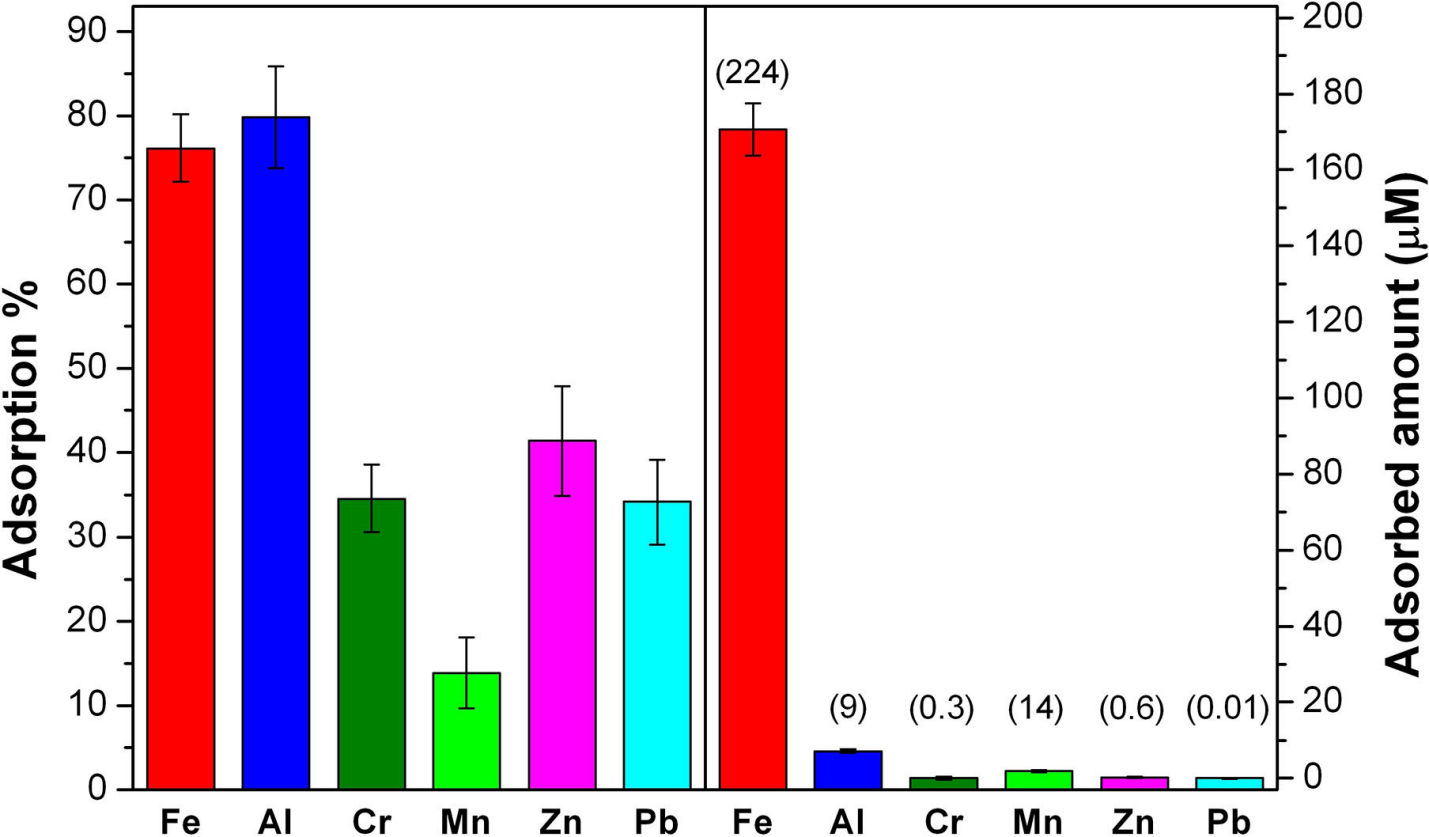
Results of the 6 h-adsorption treatment of landfill leachate at its natural pH 5.6 with SBH-A. The left panel reports the adsorption % for each metal ion. On the right panel, the removed concentration (μM) of each ion is shown and the labels are referred to the starting concentration (μM) of each ion.

In the case of the landfill leachate, it is important to note that the matrix was constituted also by organic molecules, creating a competition among the multiple components and then influencing SBH-A performances toward metallic ions. Nevertheless, the results showed in Figure 7 are encouraging since SBH-A hulls maintained their sequestrating ability, in particular toward Fe and Al, since more than 75% of these ions was removed from the solution. Moreover, it is also interesting to consider that, in the case of Fe, this percentage corresponds to a concentration of 1.7 × 10^−4^ M, confirming the good adsorption levels reached by SBH-A toward iron ions in ultrapure water, as previously discussed.

## Conclusions

Soybean hulls were recovered after the extraction of soybean peroxidase, an enzyme employed as green biocatalyst. The results of hull physicochemical characterization evidenced a remarkable impact of the enzyme extraction procedure, which varied the hull surface morphology and decreased the content of intrinsically adsorbed metallic/organic substances. Such treated hulls were applied as adsorbents of metal ions [Fe(III), Al(III), Ni(II), Mn(II), Cr(III)] in different aqueous matrixes, revealing an improvement of their sequestrating capability with respect to untreated samples. In conclusion, such residual hulls deriving from agro-industrial scraps, not only can be successfully processed to obtain a high-value enzyme, but it is also possible to extend their exploitation in water remediation field, further decreasing their environmental impact as waste and giving them an additional technological and economical value.

## Data Availability Statement

The original contributions presented in the study are included in the article/supplementary material, further inquiries can be directed to the corresponding author.

## Author Contributions

MT, MM, GM, and EL contributed conception and design of the study. MT, VT, and RS performed the experiments. MT wrote the first draft of the manuscript. GM and EL wrote sections of the manuscript. All authors contributed to manuscript revision, read, and approved the submitted version.

## Conflict of Interest

The authors declare that the research was conducted in the absence of any commercial or financial relationships that could be construed as a potential conflict of interest.
